# Cocaine and Morphine Converge to Disrupt Chloride Homeostasis in Ventral Tegmental Area GABA Neurons

**DOI:** 10.1111/adb.70104

**Published:** 2025-12-04

**Authors:** Anna C. Pearson, Blake A. Kimmey, Madison B. Taormina, William M. Holden, Alexey Ostroumov

**Affiliations:** ^1^ Department of Pharmacology & Physiology Georgetown University Washington DC USA; ^2^ Interdisciplinary Program in Neuroscience Georgetown University Washington DC USA; ^3^ Department of Neuroscience, Mahoney Institute for Neurosciences, Perelman School for Medicine University of Pennsylvania Philadelphia Pennsylvania USA

**Keywords:** addiction, dopamine, KCC2, opioids, psychostimulants, self‐administration

## Abstract

Identifying shared neural mechanisms influenced by diverse classes of drugs of abuse is essential for understanding addiction and for developing broad‐spectrum treatments for substance use disorders. Previous studies indicate that many drugs of abuse increase dopamine output from the ventral tegmental area (VTA) by altering the balance of excitatory and inhibitory inputs onto dopamine neurons, thereby promoting maladaptive plasticity within reward circuits. Here, we demonstrate in rats that acute injections of morphine and cocaine, but not saline, disrupt chloride homeostasis in VTA GABA neurons. This disruption is characterised by a depolarised GABA_A_ reversal potential, impaired chloride extrusion, and posttranslational downregulation of the potassium chloride cotransporter KCC2. Although previous studies linked drug‐induced posttranslational downregulation of KCC2 in the VTA to glucocorticoid receptor activation, we found that a glucocorticoid receptor antagonist did not prevent cocaine‐ and morphine‐induced disruption of chloride homeostasis. Instead, our data show that dopamine D1/D5 receptor activation is both necessary and sufficient for these alterations. Notably, chloride homeostasis remains impaired for several weeks after volitional morphine self‐administration, indicating long‐lasting plasticity. These findings complement previous work on nicotine and alcohol, suggesting a shared mechanism of inhibitory plasticity in the VTA following drug exposure. Given that chloride dysregulation in VTA GABA neurons influences downstream circuit function and promotes maladaptive behaviours associated with drug use, we propose KCC2 as a promising therapeutic target for substance use disorders.

## Graphical abstract

Drug exposure alters VTA circuitry across drug classes. We show that acute morphine and cocaine, but not saline, disrupt chloride homeostasis in VTA GABA neurons, producing a depolarized GABA_A_ reversal potential, impaired chloride extrusion, and posttranslational downregulation of KCC2. A glucocorticoid receptor antagonist did not prevent these effects; instead, dopamine D1/D5 receptor activation was necessary and sufficient. Chloride dysregulation persisted after volitional morphine intake, suggesting lasting inhibitory plasticity and highlighting KCC2 as a potential therapeutic target.

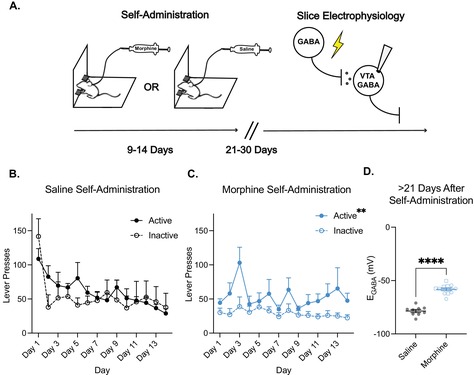

## 1 Introduction

Identifying common neural adaptations induced by different addictive substances is critical for understanding the mechanisms of addiction and for developing broadly effective treatments for substance use disorders. While individual drugs may act through distinct molecular targets, they often converge on shared neural circuits, particularly those governing reward, motivation, and learning. Understanding how addictive substances alter these circuits can reveal universal mechanisms of maladaptive plasticity, offering a unifying framework for intervention strategies that transcend specific substances. Moreover, studying these shared forms of maladaptive plasticity deepens our understanding of how the brain's learning and memory systems are co‐opted and dysregulated by addiction, ultimately guiding the development of treatments that restore healthy circuit function and improve long‐term outcomes.

One major hypothesis posits that all drugs of abuse ultimately converge on a common mechanism: increased dopamine release from neurons originating in the ventral tegmental area (VTA), a midbrain region critical for shaping reward‐related behaviours [1, 2]. While significant research has focused on dopaminergic neurons in the VTA and the plasticity they undergo following drug exposure, much less is understood about local GABAergic neurons. These inhibitory neurons regulate dopamine neuron activity and thus play a crucial role in shaping dopamine output [3]. Further, VTA GABA neurons send long‐range projections to other brain areas, where they modulate circuit activity and influence motivated and addictive behaviours [4, 5].

Growing evidence suggests that in VTA GABA neurons, drugs of abuse alter GABA_A_ receptor‐mediated synaptic transmission by disrupting intracellular chloride (Cl^−^) homeostasis, which is maintained by the K^+^/Cl^−^ cotransporter KCC2 [6, 7, 8, 9]. In the VTA and substantia nigra, KCC2 expression has been consistently observed in GABAergic neurons, whereas immunohistochemical and electrophysiological studies have generally failed to detect KCC2 protein expression or function in dopamine neurons [6, 7, 9, 10, 11]. In VTA GABA neurons, disruptions in KCC2 function can impair Cl^−^ extrusion, leading to a depolarising shift in the GABA_A_ reversal potential (*E*_GABA_). This shift weakens GABA_A_ receptor‐mediated inhibition and can even result in paradoxical GABAergic excitation of VTA GABA neurons. Disruption of KCC2, induced by stress, chronic pain, or exposure to addictive drugs, impairs inhibitory control over dopamine neurons and has been shown to contribute to drug intake and addictive behaviours [4, 6, 7, 8, 9, 12, 13].

Despite previous evidence that nicotine, alcohol, and morphine downregulate KCC2 expression in the VTA, it remains unclear whether Cl^−^ homeostasis is similarly disrupted by all addictive drugs. Furthermore, the mechanisms underlying KCC2 impairment are not fully understood. The VTA is influenced by multiple neuromodulatory systems that are robustly activated by different experiences and contribute to long‐lasting behavioural adaptations. While corticosteroid‐induced posttranslational modifications can reduce KCC2 function following acute stress [6], studies of drugs of abuse report neuroimmune‐related reductions in total KCC2 protein expression [9]. Understanding the precise regulatory pathways underlying drug‐induced KCC2 downregulation is critical for elucidating addiction mechanisms and for developing new treatments. Nevertheless, whether pharmacologically distinct drug classes converge on shared mechanisms to disrupt KCC2 function remains an important open question.

In the current study, we demonstrate that substances representing two distinct classes of drugs, opioids and stimulants, produce a common depolarising effect on *E*_GABA_ and impair Cl^−^ extrusion in VTA GABA neurons. In contrast to previous studies of chronic drug exposure [7, 9], we find that acute cocaine and morphine disrupt Cl^−^ homeostasis via posttranslational modifications in KCC2, rather than through reductions in total protein expression. These changes are triggered by activation of dopamine D1/D5 receptors but not by stress hormones. Additionally, we report long‐lasting *E*_GABA_ depolarisation in VTA GABA neurons following volitional morphine self‐administration, suggesting that impaired Cl^−^ homeostasis can contribute to the persistence of maladaptive behaviours associated with substance use disorders. Collectively, these findings support the possibility of targeting KCC2 to treat a range of substance use disorders.

## 2 Methods

### 2.1 Animals

Adult (8–16 weeks old) and juvenile (21–28 days old) male and female Long‐Evans rats (Harlan‐Envigo) were used in this study. Animals were housed in a quiet, temperature‐ and humidity‐controlled facility under a 12/12‐h light/dark cycle. Rats were group housed and had ad libitum access to food and water. All rats were handled for at least 5 days prior to the start of behavioural testing. All procedures were conducted in accordance with the Georgetown University Institutional Animal Care and Use Committee (IACUC) guidelines.

### 2.2 Surgery

All surgeries were performed under isoflurane gas anaesthesia. For VTA GABA neuron identification, adult male and female GAD‐Cre rats [14] were injected bilaterally with AAV5‐pCAG‐Flex‐EGFP‐WPRE (Addgene, Plasmid #51502) in the VTA at the following coordinates: anterior–posterior (AP) = 5.5, medial‐lateral (ML) = ±1.0, dorsal–ventral (DV) = −8.1 [15].

For self‐administration experiments, adult male rats underwent jugular catheterisation. A polyurethane catheter was surgically implanted by threading it subcutaneously over the shoulder blade and inserting it into the jugular vein, where it was secured with sutures. The catheter was connected to a mesh back‐mount platform (Instech Laboratories), which was positioned beneath the skin between the shoulder blades and sutured in place. To maintain patency, catheters were flushed daily with 0.2 mL of timentin (0.93 mg/mL, Fisher Scientific), dissolved in heparinised saline (1% heparin, Med‐Vet International) and sealed with aluminium obturators when not in use.

### 2.3 Acute Intraperitoneal Drug Injection Procedures

Acute injections of morphine sulphate (10 mg/kg, Spectrum Chemical and the NIDA Drug Supply Program) or cocaine (10 mg/kg, Sigma‐Aldrich) were given 12–15 h prior to ex vivo electrophysiology experiments. Both morphine and cocaine were dissolved in sterile saline. RU486 (40 mg/kg, Sigma‐Aldrich) was dissolved in DMSO, and SCH23390 (0.5 mg/kg, Sigma‐Aldrich) was dissolved in saline. RU486 and SCH23390 were administered i.p. 10 min prior to cocaine or morphine injections.

### 2.4 Intravenous Morphine Self‐Administration

Seven days after jugular catheterisation, adult male rats were trained to self‐administer morphine (0.25 mg/kg/infusion, *n* = 2; or 0.75 mg/kg/infusion, *n* = 9) or saline on a fixed ratio 1 (FR1) schedule. The 0.75‐mg/kg/infusion dose was selected as a standard high dose based on the morphine self‐administration literature [16, 17]. The 0.25‐mg/kg/infusion dose, a standard low dose, was included as an additional experimental cohort because at this dose, animals' total daily intake (~10 mg/kg) matched the dose used in the acute experimenter‐administered injection paradigm. Sessions occurred during the animals' active cycle (18:00–06:00). Rats receiving 0.75 mg/kg/infusion were trained for up to 12 h per day for 14 consecutive days, while rats receiving 0.25 mg/kg/infusion were trained for 6 h per day for 9 consecutive days. To prevent overdose, sessions were terminated after animals reached 75 infusions of morphine (applied to both dose groups). Five‐second infusions of morphine or saline were delivered immediately after pressing the active lever, accompanied by cue light illumination, and followed by a 20‐s ‘time‐out’ period. An inactive lever was also present; presses on it were tabulated but had no consequences.

### 2.5 Electrophysiology

Horizontal slices (220 μm) containing the VTA were cut using a vibratome (Leica Microsystems) from both juvenile and adult male and female rats. Brains were sliced in ice‐cold, oxygenated (95% O₂, 5% CO₂) high‐sucrose artificial cerebrospinal fluid (ACSF) containing 205.0 mM sucrose, 2.5 mM KCl, 21.4 mM NaHCO₃, 1.2 mM NaH₂PO₄, 0.5 mM CaCl₂, 7.5 mM MgCl₂, and 11.1 mM dextrose. Immediately after slicing, brain sections were transferred into standard ACSF consisting of 120.0 mM NaCl, 3.3 mM KCl, 25.0 mM NaHCO₃, 1.2 mM NaH₂PO₄, 2.0 mM CaCl₂, 1.0 mM MgCl₂, 10.0 mM dextrose, and 20.0 mM sucrose. Slices were continuously oxygenated (95% O₂, 5% CO₂), maintained at 32°C for 40 min and then left to equilibrate at room temperature for at least an additional hour.

**FIGURE 1:**
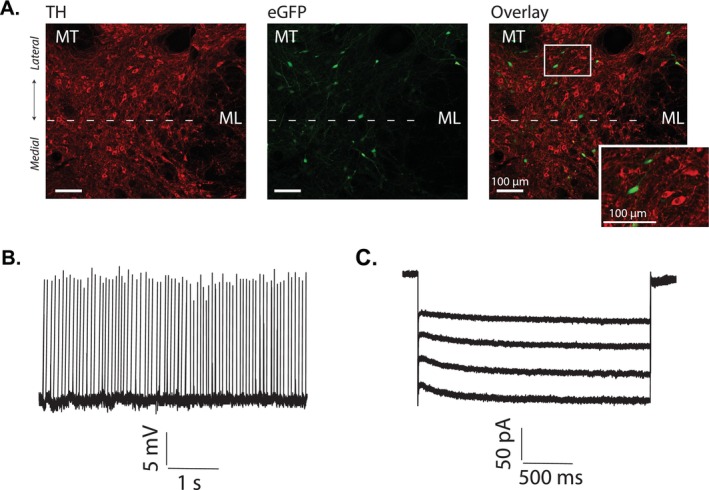
VTA GABA neurons were identified either by Cre‐dependent GFP labelling or by electrophysiological properties. (A) The lateral VTA was localised based on neighbouring landmarks, positioned medial to the medial terminal nucleus of the accessory optic tract (MT), and rostrolateral to the crest of the medial lemniscus (ML). In GAD‐Cre–expressing animals, cells virally labelled with Cre‐dependent eGFP (green) in the VTA were consistently immuno‐negative for TH (red). (B) Spontaneous firing activity was measured in cell‐attached, zero‐current mode. Under these conditions, VTA GABA neurons consistently exhibited rapid firing, with frequencies greater than 7 Hz. A representative trace illustrates this high baseline activity. (C) Hyperpolarisation‐activated currents (*I*_*h*_) were assessed in whole‐cell configuration. Voltage steps from −40 to −110 mV (10‐mV increments, 1.5 s each) were applied. The traces shown correspond to steps between −80 and −110 mV. VTA GABA neurons displayed minimal *I*_*h*_, with peak amplitudes below 150 pA.

To identify GABA neurons in the lateral VTA, a subset of GAD‐Cre rats received midbrain injections of a Cre‐dependent GFP virus. Immunohistochemistry confirmed that GFP‐labelled cells did not express tyrosine hydroxylase (TH) (Figure 1A). In wild‐type rats, lateral VTA GABA neurons were distinguished based on morphological and electrophysiological features consistent with previous reports [6, 18, 19, 20]. These neurons typically exhibit small somatic dimensions (less than 20 μm in diameter) and display a relatively fast spontaneous firing rate exceeding 7 Hz under zero current conditions in the cell‐attached mode (Figure 1B). Additionally, they show minimal hyperpolarisation‐activated current (*I*_*h*_), with peak amplitudes below 150 pA in whole‐cell recordings (Figure 1C). In our previous studies, we showed that cells with these properties were consistently tyrosine hydroxylase (TH) negative [6, 8]. Importantly, the GFP‐positive cells labelled in GAD‐Cre rats exhibited electrophysiological properties identical to those of the putative GABA neurons recorded in wild‐type rats, further confirming their identity as GABAergic neurons.

To record *E*_GABA_ from VTA GABA neurons, perforated‐patch recordings were used. Gramicidin was first dissolved in methanol (10 mg/mL), then diluted into the pipette solution (135.0 mM KCl, 12.0 mM NaCl, 10.0 mM HEPES, 0.5 mM EGTA, 0.3 mM Tris‐GTP, 2.0 mM Mg‐ATP, pH 7.2–7.3) to a final concentration of 150 μg/mL. Synaptic stimulation was delivered using a bipolar tungsten electrode (World Precision Instruments) positioned 100–150 μm from the recording site. GABA_A_ receptor‐mediated inhibitory postsynaptic currents (IPSCs) were isolated using 20 μM DNQX, 50 μM AP5, and 1 μM CGP55845. IPSCs were evoked and recorded under voltage clamp at various holding potentials, and *E*_GABA_ was determined from the current–voltage relationship. To prevent action potential generation at depolarised holding potentials, tetrodotoxin (0.5 μM, HelloBio) was added to the extracellular solution. For studies assessing the impact of D1/D5 receptor activation on *E*_GABA_, slices were incubated in SKF81297 (10 μM, Sigma‐Aldrich) for 15 min.

To assess activity‐dependent synaptic depression, whole‐cell voltage‐clamp recordings were made during repeated 20 Hz synaptic stimulation. The internal solution used for these experiments included 123.0 mM K⁺ gluconate, 8.0 mM NaCl, 2.0 mM Mg‐ATP, 0.2 mM EGTA, 10.0 mM HEPES, and 0.3 mM Tris‐GTP (pH 7.2–7.3). GABA_A_‐mediated IPSCs were isolated with 20 μM DNQX, 50 μM AP5, and 1 μM CGP55845.

Electrophysiological recordings were acquired using an Axopatch 200B amplifier (Molecular Devices) and digitised at 20 kHz using pClamp 9.2 (Digidata Interface, Molecular Devices). *E*_GABA_ recordings were low‐pass filtered at 10 kHz. Data were analysed offline using Clampfit 11.2.

### 2.6 Immunohistochemistry and Imaging

TH labelling was performed on midbrain slices of GAD‐Cre rats injected with Cre‐dependent GFP. For transcardial perfusion, deep anaesthesia was induced with isoflurane, after which animals were perfused with 50 mL phosphate‐buffered saline (PBS; EMD Millipore), followed by 40 mL of 4% paraformaldehyde (PFA; Chembiotec). Brains were extracted, postfixed in 4% PFA for 2 h, and cryoprotected by immersion in ascending sucrose solutions (10%, 20%, 30% in PBS) until saturation. Tissue was then embedded in OCT, frozen, and stored at −80°C. Horizontal sections of the VTA (40 μm) were cut and maintained as free‐floating slices in PBS.

Sections were incubated overnight at 4°C with a primary antibody against TH (1:1000; Invitrogen). After PBS washes, slices were exposed for 3 h at room temperature to the fluorescent secondary antibody goat anti‐rabbit Alexa Fluor 594 (1:1000; Invitrogen). Immunolabelled sections were imaged with a Mica Microhub (Leica Microsystems) confocal microscope. All illumination settings were automatically adjusted using the OneTouch function in LAS X software (Leica Microsystems), and exported images were further processed with Fiji software (ImageJ).

### 2.7 Western Blots

The VTA was dissected from horizontal brain slices of adult rats, with slice preparation conducted as outlined in the Electrophysiology section. Membrane fractions were isolated using the Mem‐PER Plus Membrane Protein Extraction Kit (Model #89842; Thermo Scientific, Rockford, IL, USA). Protein samples (30 μg) in 2.5% 2‐mercaptoethanol were separated on a 4%–15% Precast Protein Gel (#4561083; Bio‐Rad) and transferred to a nitrocellulose membrane (Bio‐Rad). Primary antibodies included rabbit anti‐KCC2 antibody (1:400, #07‐432; Millipore, Temecula, CA, United States) and mouse anti‐GAPDH antibody (1:400, #MAB374; Millipore). Secondary antibodies were goat anti‐rabbit IgG (#T2191; Applied Biosystems, Foster City, CA, United States) or goat anti‐mouse IgG/IgM (#T2192; Applied Biosystems), with all antibodies diluted in SignalBoost solution (#407207; EMD Millipore, Billerica, MA, United States). Membranes were developed using Tropix CDP‐Star solution (T2218; Applied Biosystems) for 5 min and then scanned with the Protein Simple FluorChem R chemiluminescence detector. A densiometric analysis was performed using the AlphaView SA software. Optical densities of KCC2‐specific bands were measured and normalised to GAPDH as the loading control.

### 2.8 Statistical Analyses

Statistical analyses were performed using GraphPad Prism (version 10). Two‐tailed *t*‐tests or one‐way ANOVA tests were conducted to compare group means for GABA reversal potential. For Western blot experiments, paired *t*‐tests were used to compare protein expression levels between saline‐ and drug‐treated animals from the same cage. Repeated‐measures two‐way ANOVA analyses were used to evaluate data from repetitive synaptic stimulation assays and morphine self‐administration. Statistical significance was defined as *p* < 0.05 for all comparisons. ANOVAs were followed with appropriate post hoc tests (with correction for multiple comparisons) when warranted. All data are presented as mean ± SEM.

## 3 Results

### 3.1 Acute Cocaine and Morphine Impair Cl^−^ Homeostasis in VTA GABA Neurons

**FIGURE 2:**
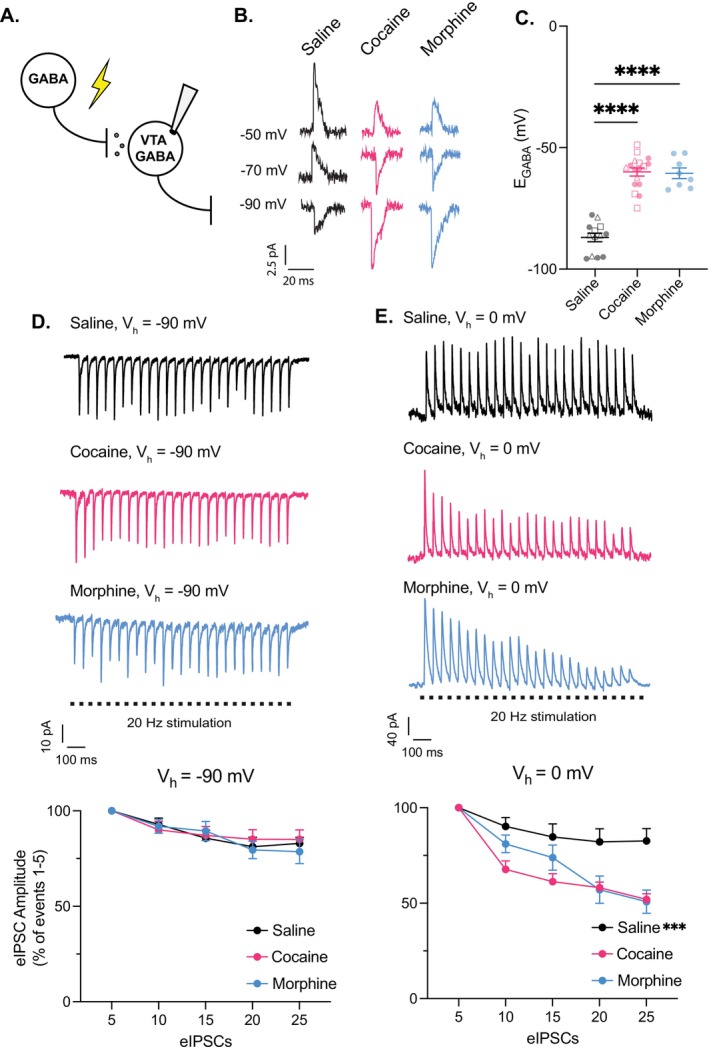
Acute cocaine and morphine impair Cl^−^ homeostasis in VTA GABA neurons. (A) To assess cocaine‐ and morphine‐induced alterations in anion homeostasis, GABAergic input onto VTA GABA neurons was recorded using gramicidin‐perforated patch‐clamp at various holding potentials. IPSCs were elicited through electrical stimulation in the presence of DNQX, AP5, CGP55845, and TTX to isolate GABAergic currents. (B) Representative traces of IPSC recordings from saline‐ (black), cocaine‐ (pink), and morphine‐treated (blue) rats at the given holding potentials. The IPSCs reverse direction at the *E*_GABA_. The traces were filtered, and stimulus artefacts were removed. (C) One‐way ANOVA revealed a significant effect of treatment on *E*_GABA_ (*F*_(2,35)_ = 72.11, *p* < 0.0001). Tukey's post hoc test showed that both cocaine‐ (−60.02 ± 1.60 mV, pink) and morphine‐treated neurons (−60.56 ± 2.18 mV, blue) were significantly more positive than saline (−86.98 ± 1.74 mV, black, *p* < 0.0001), with no difference between cocaine and morphine groups (*p* > 0.05). No significant differences by sex or age were detected within cocaine groups (adult males shown as open squares; adult females shown as open triangles; juveniles shown as closed circles, unpaired *t*‐tests, *p* > 0.05, *n* = 2–8 cells/group). (D) To assess activity‐dependent synaptic depression, whole‐cell patch‐clamp recordings were performed on VTA GABA neurons during repeated activation of GABA_A_ receptors. Top traces show representative recordings from saline‐ (black), cocaine‐ (pink), and morphine‐exposed (blue) animals, both exhibiting comparable changes in evoked IPSC (eIPSC) amplitude during 20 Hz stimulation at a holding potential of −90 mV. Bottom panel quantifies this effect, showing that the rate of eIPSC amplitude decline did not significantly differ between groups (repeated measures two‐way ANOVA, group × time, *F*(8, 92) = 0.6853, *p* = 0.70, *n* = 9 cells/group, *n* = 4 rats/group). Amplitudes were averaged in sets of five and expressed as a percentage of the mean amplitude of the initial five responses. (E) To examine activity‐dependent intracellular Cl^−^ accumulation in VTA GABA neurons, a similar protocol was used, but cells were voltage‐clamped at 0 mV. Top traces illustrate a modest reduction in eIPSC amplitude in a GABA neuron from a control animal (black) compared with a more pronounced depression in neuron from cocaine‐ (pink) and morphine‐treated (blue) rats. Bottom panel shows that at 0 mV, GABA neurons from cocaine‐ and morphine‐exposed animals exhibited a significantly enhanced synaptic depression relative to saline‐treated controls (repeated measures two‐way ANOVA, group × time, *F*_(8,92)_ = 4.935, *p* < 0.0001, *n* = 9 cells/group, *n* = 4 rats/group). Šídák's multiple comparisons test confirmed greater synaptic depression in cocaine‐ and morphine‐exposed neurons compared with saline over time (*p* < 0.05).

*E*_GABA_ represents the membrane potential at which evoked inhibitory postsynaptic currents (IPSCs) reverse direction from inward to outward. To determine *E*_GABA_ in VTA GABA neurons, we conducted gramicidin‐perforated patch‐clamp recordings, which preserve intracellular anion concentrations, and measured GABA_A_ receptor‐mediated inhibitory postsynaptic currents (IPSCs) across different membrane potentials (Figure 2A). *E*_GABA_ was recorded following a single injection of cocaine (10 mg/kg, i.p.), morphine (10 mg/kg, i.p.), or saline 12–15 h before slice preparation. VTA GABA neurons from cocaine and morphine‐treated rats exhibited a significantly more depolarised *E*_GABA_ compared with saline‐treated rats (Figures 2B,C). Notably, further analysis after cocaine exposure revealed no significant differences between sexes or age groups (see Figure 1C legend for details).

Depolarising shifts in *E*_GABA_ result from elevated intracellular Cl^−^ levels, often due to impaired Cl^−^ extrusion. During prolonged GABA_A_ receptor activation, reduced Cl^−^ extrusion leads to intracellular Cl^−^ accumulation, ultimately weakening synaptic inhibition by diminishing the Cl^−^ gradient. To assess whether acute cocaine or morphine exposure compromises Cl^−^ extrusion in VTA GABA neurons, we applied repetitive GABA_A_ receptor stimulation and measured activity‐dependent IPSC depression [6, 21, 22]. The rate of IPSC amplitude reduction at 0 mV, where Cl^−^ influx dominates, reflects Cl^−^ accumulation and activity‐dependent synaptic depression. In contrast, at −90 mV, where Cl^−^ efflux prevails, synaptic depression occurs without accompanying Cl^−^ accumulation.

Following 20 Hz electrical stimulation at −90 mV, cocaine and morphine had no effect on the rate of synaptic depression in VTA GABA neurons (Figure 2D). However, at 0 mV, IPSC amplitude declined significantly faster in cocaine‐treated animals compared with saline‐treated controls (Figure 2E). The distinct effects of acute cocaine and morphine exposure at −90 mV versus 0 mV indicate increased intracellular Cl^−^ accumulation, suggesting a diminished Cl^−^ extrusion capacity in VTA GABA neurons.

### 3.2 Acute Cocaine and Morphine Decrease KCC2 Phosphorylation at Ser940 in VTA

**FIGURE 3:**
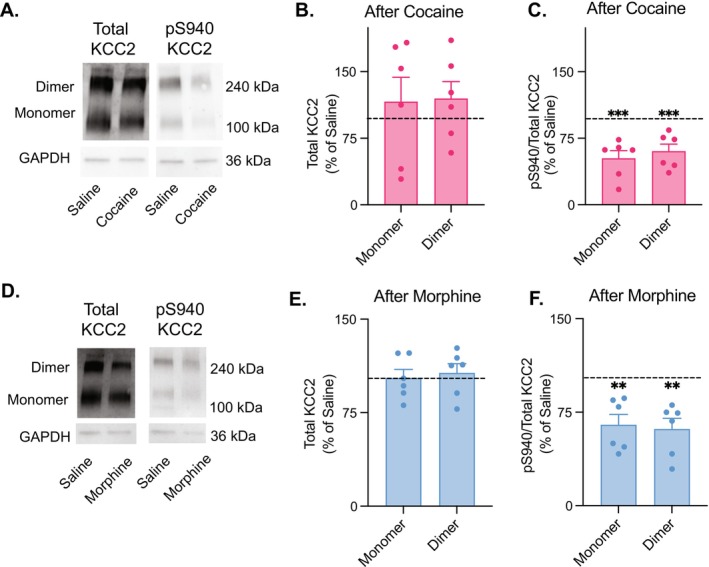
Acute cocaine and morphine decrease KCC2 phosphorylation at Ser940 in VTA. (A) Western blot analysis was performed to assess total KCC2 and phosphorylated‐S940 KCC2 levels, using GAPDH as a loading control. A representative blot revealed no changes in total KCC2 expression following cocaine. However, cocaine‐treated animals exhibited a reduction in pS940 KCC2 relative to total KCC2 compared with saline‐treated controls. (B) Densitometric analysis did not reveal a significant decrease in total KCC2 expression in cocaine‐treated animals compared with saline‐treated controls (horizontal dashed line at 100% represents the control mean): paired *t*‐tests, 116.0% ± 27.64%, *p* = 0.59 for monomers, 119.6% ± 19.19%, *p* = 0.35 for dimers, *n* = 6 rats/group, normalised to saline controls. (C) Densitometric analysis showed a significant decrease in the pS940 KCC2 to total KCC2 ratio in cocaine‐treated animals compared with saline‐treated controls (horizontal dashed line): paired *t*‐tests, 52.28% ± 8.76%, *p* = 0.0028 for monomers, 60.31% ± 8.15%, *p* = 0.0046 for dimers, *n* = 6 rats/group, normalised to saline controls. (D) Immunoblotting was used to evaluate expression levels of total KCC2 and its phosphorylated form at serine 940 (pS940), with GAPDH serving as the loading control. Representative blots showed that total KCC2 protein levels remained unchanged following morphine exposure. In contrast, morphine‐treated animals displayed a decreased ratio of pS940 to total KCC2 compared with saline controls, indicating reduced phosphorylation of KCC2 at this regulatory site. (E) Quantification of band intensity revealed no significant difference in total KCC2 protein levels between morphine‐ and saline‐treated groups (horizontal dashed line): paired *t*‐tests, 102.7% ± 6.97%, *p* = 0.71 for monomers, 106.8% ± 7.57%, *p* = 0.41 for dimers, *n* = 6 rats/group, normalised to saline controls). (F) In contrast, morphine exposure led to a significant reduction in the ratio of phosphorylated S940‐KCC2 to total KCC2, as determined by densitometric analysis (horizontal dashed line): paired *t*‐tests, 64.81% ± 8.44% for monomers, *p* = 0.0087, 61.47% ± 8.54% for dimers, *p* = 0.0063, *n* = 6 rats/group, normalised to saline controls).

Drug‐induced disruption of Cl^−^ homeostasis has previously been linked to reduced expression of the K^+^, Cl^−^ co‐transporter KCC2 [6, 7, 8, 9, 23, 24]. However, stress‐induced depolarising shifts in *E*_GABA_ within the VTA have been associated with the decreased phosphorylation at serine 940, rather than changes in total KCC2 protein expression. To investigate whether acute cocaine and morphine administration alters KCC2 expression or phosphorylation within the VTA, we performed Western blot analyses using antibodies against total KCC2 and phosphorylated KCC2 at serine 940 (pS940‐KCC2). Immunoblotting revealed prominent bands at 140 and 270 kDa for both total and pS940‐KCC2, corresponding to the presence of monomeric and dimeric forms of KCC2 (Figures 3A,D). Cocaine exposure did not significantly change total KCC2 levels (Figure 3B). However, the ratio of pS940‐KCC2 to total KCC2 was significantly reduced in cocaine‐treated animals (Figure 3C). Additionally, we did not find a significant difference in total KCC2 protein expression after morphine exposure (Figure 3E), although the ratio of phosphorylated S940‐KCC2 to total KCC2 was significantly reduced following the acute morphine injection (Figure 3F). These findings suggest a posttranslational mechanism underlying the observed functional impairment of VTA GABA cells following acute cocaine and morphine exposure.

### 3.3 Cocaine and Morphine‐Mediated *E*_GABA_ Depolarisation in VTA GABA Neurons Is not Mediated Through Stress Hormones

In the VTA, drug‐induced dephosphorylation of KCC2 at serine 940 has previously been associated with stress [6, 7, 8]. For example, acute nicotine exposure leads to a rapid increase in plasma corticosterone, which in turn impairs *E*_GABA_ and inhibitory synaptic transmission in VTA GABA neurons, with effects lasting from hours to days [8, 25]. Given that both acute cocaine and morphine have been shown to elevate stress hormones [26, 27, 28, 29], we next investigated whether KCC2 downregulation following cocaine or morphine exposure was triggered by stress signalling. Specifically, we tested whether cocaine‐ and morphine‐induced *E*_GABA_ depolarisation could be prevented by the glucocorticoid receptor antagonist RU486, which has been shown to block stress and nicotine‐induced impairment of Cl^−^ homeostasis [6, 7].

**FIGURE 4:**
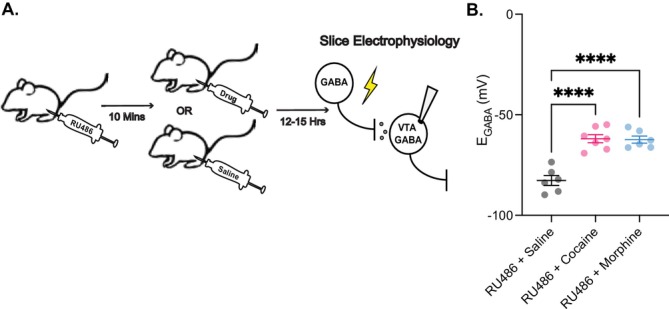
Cocaine and morphine‐mediated *E*_GABA_ depolarisation in VTA GABA neurons is not triggered by stress hormones. (A) Rats received an i.p. injection of RU486, a glucocorticoid receptor antagonist, 10 min before receiving a second i.p. injection of either cocaine, morphine, or saline. 12–15 h later, rats were sacrificed, and brains were prepared for slice electrophysiology. (B) Injections of RU486 did not prevent the depolarisation of *E*_GABA_ in VTA GABA cells from cocaine‐ or morphine‐treated animals compared with saline controls (RU486 + cocaine: −61.89 ± 2.03 mV, *n* = 7 cells, 4 rats; RU486 + morphine: −62.32 ± 1.77 mV, *n* = 6 cells, 4 rats; RU486 + saline: −82.65 ± 2.47 mV, *n* = 6 cells, 4 rats). One‐way ANOVA revealed a significant effect of treatment (*F*_(2,16)_ = 30.98, *p* < 0.0001). Tukey's post hoc test showed that both RU486 + cocaine and RU486 + morphine groups differed significantly from RU486 + saline (*p* < 0.0001), whereas RU486 + cocaine and RU486 + morphine did not differ from each other (*p* > 0.05).

We performed systemic injections of RU486 10 min prior to cocaine or morphine exposure and measured *E*_GABA_ 12–15 h after (Figure 4A). RU486 did not prevent the drug‐induced depolarising shift in *E*_GABA_ in VTA GABA neurons (Figure 4A,B), suggesting that the observed effects are not mediated by stress‐related pathways.

### 3.4 Cocaine and Morphine‐Mediated *E*_GABA_ Depolarisation in VTA GABA Neurons is D1/D5 Receptor‐Dependent

**FIGURE 5:**
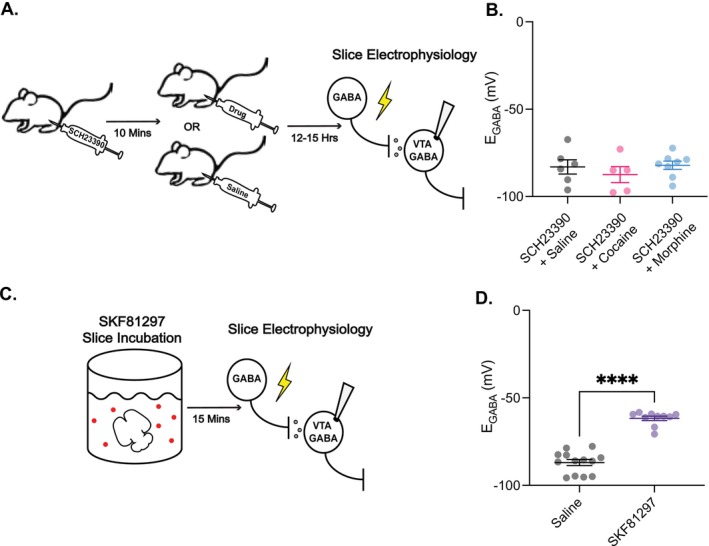
Cocaine and morphine‐mediated *E*_GABA_ depolarisation in VTA GABA neurons is D1/D5 receptor‐dependent. (A) Rats were administered an i.p. injection of the selective D1/D5 receptor antagonist SCH23390 followed 10 min later by a second injection of either cocaine, morphine, or saline. After 12–15 h, animals were euthanised, and brain tissue was collected for ex vivo slice electrophysiology. (B) Injections of SCH23390 prevented the depolarisation of *E*_GABA_ in VTA GABA cells in cocaine and morphine treated animals versus saline treated animals: −87.46 ± 4.56 mV for SCH23390 + cocaine (*n* = 5 cells, 2 animals; pink data), −82.10 ± 2.37 mV for SCH23390 + morphine (*n* = 8 cells, 4 animals; blue data), versus −83.02 ± 4.07 mV for SCH23390 + saline (*n* = 6 cells, 4 animals; black data), one‐way ANOVA, *F*_(2,16)_= 0.61, *p* = 0.56. (C) Brain slices from naïve rats were incubated in the D1/D5 receptor agonist SKF81297 for 15 min before cells were recorded. (D) Slice incubation SKF81297 resulted in the depolarisation of *E*_GABA_ in VTA GABA neurons in naïve animals; −61.68 ± 1.24 mV for SKF81297‐treated cells versus −88.65 ± 3.16 mV (*n* = 10 cells, 6 animals; purple data) for control cells (*n* = 13 cells, 7 animals; black data), unpaired *t*‐test, *p* < 0.0001.

Besides activation of the stress system, another shared characteristic of both cocaine and morphine is their ability to elevate dopamine levels within the brain's reward circuitry [30, 31]. Because dopamine release acting through D1/D5 receptor activation facilitates synaptic plasticity in the VTA [32, 33], we next investigated whether D1/D5 receptor activation plays a role in the depolarising shift of *E*_GABA_. First, we systemically injected the selective D1/D5 receptor antagonist SCH23390 10 min before cocaine or morphine exposure. Then, *E*_GABA_ was recorded 12–15 h after drug exposure (Figure 5A). While SCH23390 alone did not alter *E*_GABA_ in VTA GABA neurons, it completely blocked cocaine‐ and morphine‐induced depolarising shift in *E*_GABA_ (Figure 5B).

Next, we incubated brain slices from drug‐naïve animals in the D1/D5 receptor agonist SKF81297 for 15 min before recording from VTA GABA neurons (Figure 5C). In contrast to control slices, incubation of brain slices from drug‐naïve animals with SKF81297 mimicked the effects of cocaine and morphine, inducing a comparable depolarisation of *E*_GABA_ in VTA GABA neurons (Figure 5D). Overall, these results demonstrate that D1/D5 receptor activation is necessary and sufficient for the cocaine‐ and morphine‐mediated *E*_GABA_ depolarisation in VTA GABA neurons.

### 3.5 Morphine Self‐Administration Leads to a Long‐Term Disruption of Cl^−^ homeostasis

**FIGURE 6:**
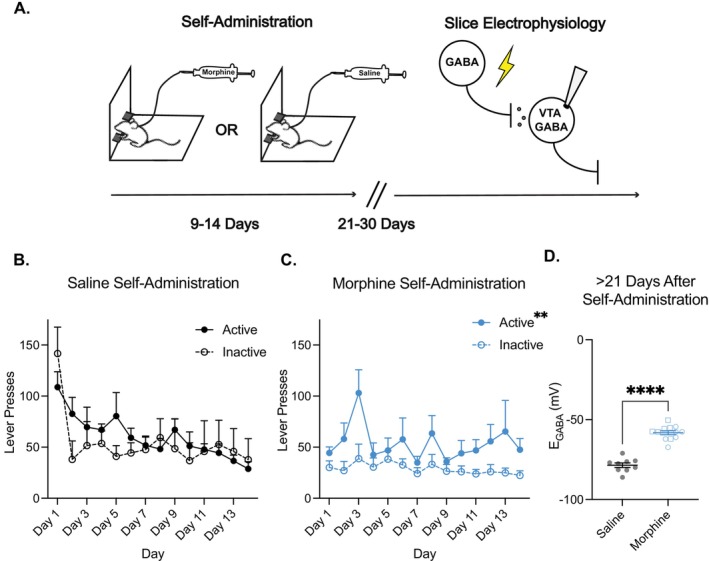
Morphine self‐administration leads to a long‐term disruption of Cl^−^ homeostasis. (A) Rats were trained to self‐administer morphine (0.25 mg/kg/infusion or 0.75 mg/kg/infusion) or saline for 12 h per day, over 14 consecutive days. 21–30 days following the final self‐administration session, rats were sacrificed for electrophysiology experiments. (B) Saline‐administering rats (*n* = 5) did not develop a preference for the active lever over the course of 14 self‐administration sessions (two‐way ANOVA, effect of lever, *F*_(1,8)_ = 0.16, *p* = 0.6999). (C) Morphine self‐administering rats (0.75 mg/kg/infusion, *n* = 9) demonstrated a preference for the active lever (two‐way ANOVA, main effect of lever, *F*_(1,20)_ = 9.38, *p* = 0.0061). (D) Morphine self‐administration (0.25 or 0.75 mg/kg/infusion) resulted in a long‐lasted depolarisation of *E*_GABA_ in VTA GABA neurons compared with saline self‐administration: −58.22 ± 1.29 mV for morphine self‐administering animals (*n* = 12 cells, 4 animals; blue data) versus −78.53 ± 1.44 mV for saline self‐administering animals (*n* = 8 cells, 3 animals; black data), unpaired *t*‐test, *p* < 0.0001. There was no significant difference in *E*_GABA_ between rats that self‐administered 0.75 mg/kg/infusion (open circles: −60.01 ± 1.83 mV) and those that received 0.25 mg/kg/infusion (open squares: −56.43 ± 1.57 mV; unpaired *t*‐test, *p* = 0.16, *n* = 6 cells/group).

Compared with passive, non‐contingent drug exposure, volitional drug intake can induce differential changes within the mesolimbic system [34, 35, 36, 37, 38, 39]. To investigate the potential differences between experimenter‐induced and volitional morphine on Cl^−^ homeostasis in the VTA, we used an operant intravenous self‐administration paradigm. Rats were trained to self‐administer either morphine or saline for 9–14 consecutive days. Twenty‐one to thirty days after the final self‐administration session, animals were sacrificed and acute brain slices containing the VTA were prepared for whole‐cell electrophysiological recordings to assess changes in GABAergic signalling (Figure 6A).

Rats trained to self‐administer saline showed a comparable number of presses on the active and inactive levers (Figure 6B). In contrast, rats trained to self‐administer morphine (0.75 mg/kg/infusion) demonstrated a significant preference for the active lever over the inactive lever (Figure 6C). These animals consumed an average of 22.08 ± 2.30 mg/kg of morphine per session, which is more than twice the dose used in acute injection experiments (10 mg/kg). To match daily morphine intake with that of the acute injection paradigm, an additional cohort of rats was trained to self‐administer a lower dose (0.25 mg/kg/infusion) for 9 consecutive days (6 h per day). Their average intake was 10.59 ± 1.88 mg/kg per session.

Between 21 and 30 days after the final self‐administration session, VTA GABA neurons from morphine, but not saline, rats showed a depolarised *E*_GABA_ (Figure 6D). Notably, there were no significant differences in *E*_GABA_ between rats that self‐administered 0.75 mg/kg/infusion and those that received 0.25 mg/kg/infusion. These findings indicate a persistent disruption of Cl^−^ homeostasis following volitional morphine exposure.

## 4 Discussion

The present study identifies a convergent neural mechanism by which distinct classes of drugs of abuse, specifically cocaine and morphine, impair GABA_A_ receptor transmission and trigger inhibitory plasticity within VTA GABA neurons. Our findings demonstrate that both acute cocaine and morphine exposure lead to a common impairment of Cl^−^ homeostasis in VTA GABAergic neurons, characterised by a depolarised GABA_A_ reversal potential (*E*_GABA_) and a diminished capacity for Cl^−^ extrusion. Crucially, we show that this disruption is mediated through dopamine D1/D5 receptor activation and involves a decrease in KCC2 phosphorylation at serine 940. Furthermore, we report that volitional morphine self‐administration results in a long‐lasting impairment of Cl^−^ homeostasis in these VTA GABA neurons. These results underscore the critical role of KCC2 in maintaining inhibitory control in the VTA and suggest its modulation as a novel therapeutic target for substance use disorders.

This study is the first to show that cocaine impairs Cl^−^ extrusion in VTA GABA neurons via KCC2 dephosphorylation. However, previous studies have reported that repeated morphine injections lead to impaired GABA_A_ receptor inhibition in VTA GABA neurons [40] and BDNF‐dependent reduction in total KCC2 protein expression [9]. Until the current study, however, the impact of acute exposure to morphine on KCC2 was unknown, as well as the mechanisms behind KCC2 changes. Here, we found that a single injection of either cocaine or morphine was sufficient to impair KCC2 function, as evidenced by a depolarised *E*_GABA_ and an impaired ability to extrude Cl^−^. While total KCC2 protein expression was not significantly changed, there was a significant reduction in the ratio of phosphorylated KCC2 at serine 940 to total KCC2, implicating a posttranslational mechanism in the observed functional impairment. Phosphorylation at serine 940 is known to stabilise KCC2 at the plasma membrane, while dephosphorylation mediated by a calcium‐dependent phosphatase promotes its internalisation and functional downregulation [41]. However, it is important to acknowledge that other KCC2 regulatory mechanisms, including alternative phosphorylation sites, glycosylation, and non‐coding RNAs, as well as the involvement of other Cl^−^ transporters, may also contribute to the disruption of Cl^−^ homeostasis induced by cocaine and morphine [41, 42].

Previous studies have shown that acute stress downregulates KCC2, increasing vulnerability to alcohol consumption [6]. Similarly, acute nicotine exposure elevates corticosterone levels in rats, triggering KCC2 downregulation that persists for several days [8, 25]. However, our results show that blocking corticosterone and progesterone receptors with RU486 does not prevent cocaine‐ or morphine‐induced depolarisation of *E*_GABA_. This suggests that, unlike nicotine or stress, cocaine and morphine do not primarily rely on glucocorticoid release or hypothalamic–pituitary–adrenal (HPA) axis activation to disrupt Cl^−^ regulation.

Instead, we found that activation of dopamine D1 and D5 receptors was both necessary and sufficient to cause *E*_GABA_ depolarisation following cocaine and morphine exposure, implicating a previously unrecognised dopaminergic mechanism in KCC2 downregulation. While systemic administration of a dopamine receptor antagonist prevented drug‐induced effects on GABA neurons, this approach does not clarify whether these effects arise from dopamine signalling within the VTA itself. Alternatively, dopamine may act in other brain regions that indirectly influence VTA GABA neurons. Moreover, brain slice incubation with dopamine agonists does not fully replicate endogenous drug‐induced dopamine dynamics or the broader neuromodulatory environment present during in vivo drug exposure. Therefore, although our convergent findings strongly support a role for D1/D5 receptors, cell‐type‐specific manipulations, such as viral D1/D5 receptor knockdown or local intra‐VTA infusions, will be essential to dissect the precise mechanisms underlying drug‐induced changes in *E*_GABA_ and KCC2 function. Finally, while our pharmacological experiments implicate dopamine and exclude glucocorticoids, additional neuromodulatory systems may also contribute. Specifically, serotonergic, noradrenergic, and opioid signalling pathways remain to be explored in relation to drug‐induced KCC2 modulation.

Because prior studies examining KCC2 in the context of opiates have relied on passive drug administration models [9, 24], we sought to determine whether volitional drug intake, known to engage distinct neural adaptations [35, 39], would differentially alter *E*_GABA_. Unlike passive drug delivery, self‐administration more closely mimics the patterns and neurobiological consequences of human opioid use, including the development of drug‐taking habits, altered reward processing, and persistent adaptations in mesolimbic circuits. Our findings indicate that morphine self‐administration leads to a lasting disruption of Cl^−^ homeostasis in VTA GABA neurons. While these results demonstrate persistent physiological alterations, it remains unclear whether the changes in *E*_GABA_ arise from long‐term posttranslational modifications of KCC2, alterations in total KCC2 protein expression, or involvement of other Cl^−^ transport mechanisms. Additionally, these self‐administration experiments were conducted only in male rats and therefore do not capture potential sex‐dependent differences in *E*_GABA_ shifts following chronic drug exposure. This sustained impairment parallels the long‐lasting alterations in VTA GABAergic signalling observed after adolescent nicotine exposure, but contrasts with the transient effects of acute or two‐week experimenter‐administered nicotine in adulthood, which typically subside within days to a few weeks [7, 25].

By identifying a convergent mechanism of inhibitory dysfunction, these findings highlight KCC2 as a promising molecular entry point for restoring normal VTA circuit function across multiple drug classes. Notably, reduced KCC2 function in VTA GABA neurons may shape behaviour by altering the activity of distinct dopamine and GABA output pathways. To date, this downregulation has been shown to reduce drug‐evoked dopamine release within the mesolimbic VTA–NAc pathway, a circuit critical for reward and addictive behaviours [6, 7, 8, 9, 23, 25]. Given the functional diversity of VTA DA projections [43, 44, 45, 46, 47], future studies should explore whether KCC2 dysregulation differentially affects distinct mesolimbic subcircuits, which play dissociable roles in reward‐related behaviours [48, 49]. Beyond mesolimbic circuits, KCC2 downregulation may also impact VTA dopamine projections to cortical and amygdala regions implicated in addiction [4]. Furthermore, KCC2 can be downregulated in VTA GABA projection neurons, which directly target forebrain and brainstem regions involved in reward and aversion [50, 51, 52, 53]. Linking these cellular adaptations to changes in dopamine and GABA circuitry, motivation, withdrawal, and drug‐seeking behaviour will be a crucial next step in establishing the functional relevance of KCC2 downregulation. Thus, future studies elucidating the circuit and behavioural consequences of KCC2 dysregulation may provide important insights for developing interventions that reverse the maladaptive adaptations underlying addictive behaviours.

## Author Contributions

A. C. P. conducted the behaviour and electrophysiology experiments, analysed the data, wrote the manuscript, prepared the figures, prepared the initial manuscript draft, and contributed to manuscript editing. B. A. K. conducted the electrophysiology experiments. M. B. T. and W. M. H. conducted the Western blot experiments. A. O. conceptualised and supervised the project, acquired the funding, conducted the electrophysiology experiments, and prepared the manuscript with A. C. P.

## Funding

This study was supported by the National Institutes of Health [DA048134 (A.O.), MH125996 (A.O.), NS121780 (A.P.)], the Whitehall Foundation [2020‐12‐35 (A.O.)], the Brain Research Foundation [BRFSG‐2022‐06 (A.O.)], and the Brain and Behavior Research Foundation [28113 (A.O.)].

## Ethics Statement

This study was conducted in accordance with the Georgetown University Institutional Animal Care and Use Committee (IACUC) guidelines.

## Conflicts of Interest

The authors declare no conflicts of interest.

## Data Availability

Data available on request from the authors.
